# The developmental, genetic, and epigenetic basis of polyendocrine metabolic ovarian syndrome

**DOI:** 10.3389/fendo.2026.1924187

**Published:** 2026-10-28

**Authors:** Abdallah Mahdi, Abeer Qannan, Nady El Hajj

**Affiliations:** 1 College of Health and Life Sciences, Hamad bin Khalifa University, Doha, Qatar; 2 College of Science and Engineering, Hamad bin Khalifa University, Doha, Qatar

**Keywords:** anti-Müllerian hormone (AMH), developmental origin of health and disease (DOHaD), developmental plasticity, epigenetics, PCOS, PMOS, polyendocrine metabolic ovarian syndrome, polycystic ovarian syndrome

## Abstract

Polycystic ovary syndrome (PCOS) is the most common reproductive and endocrine disorder affecting women in their reproductive age. PCOS is characterized by clustering of reproductive disturbances, including, but not limited to, hypothalamus-pituitary-ovarian axis dysfunction, chronic anovulation, excess androgen, and subfertility. PCOS extends beyond reproductive dysfunction to involve metabolic, endocrine, and cardiometabolic abnormalities. Therefore, it has recently been redefined as polyendocrine metabolic ovarian syndrome (PMOS). Despite the evolution of diagnostic criteria for PMOS in recent decades, there persists a knowledge gap regarding its origins and pathophysiology. Genetic studies have identified candidate genes associated with PMOS, but genetic factors alone cannot account for its varied phenotypes. The diverse phenotypes observed in PMOS instead point to a complex gene-environment interaction, thereby paving the way for epigenetic mechanisms to explain this heterogeneous trait. Emerging evidence suggests that epigenetic alterations during critical developmental periods, particularly *in utero* and during puberty, may contribute to the reproductive, metabolic, and endocrine manifestations of PMOS. These observations may re-frame PMOS as a developmental disorder that follows the developmental origin of health and disease (DOHaD) theory. In this review, we propose a comprehensive framework to address the complexity of PMOS pathogenesis and phenotypes. We investigated how environmental exposures during critical windows may shape the epigenome and contribute to PMOS development. The review also examines the biological rationale of the 2023 International Evidence-based Guideline for PCOS, including the use of anti-Müllerian hormone, postponing diagnosis until after puberty, and recommendations for healthy lifestyle behaviors.

## Introduction

Since its initial description in 1935, polycystic ovary syndrome (PCOS) has evolved significantly in its understanding and diagnosis. Initially, Stein and Leventhal defined PCOS as a reproductive disorder that is distinguished by “polycystic” appearance of the ovaries and abnormal bleeding patterns (1). Obesity and clinical features of excess androgen, including hirsutism and acne, were originally observed in some patients. However, the importance of hyperandrogenism in PCOS was not fully appreciated until the 1970s-1980s, when research began to identify its effect on affected females (2, 3). In 1989, Dunaif et al. demonstrated that insulin resistance is intrinsic to PCOS rather than secondary to obesity, a finding that established a mechanistic link between PCOS and the subsequent risk of metabolic syndrome (4).

These discoveries shifted the focus from a purely reproductive disorder to a more complex multisystem condition. Ultimately, accumulating evidence led to the development of the first NIH diagnostic criteria in 1990 (5). These criteria required the presence of oligo- or anovulation and hyperandrogenism to diagnose PCOS (5). Some authors have referred to PCOS as a ‘disease’ to emphasize its significant morbidity and a better understanding of its molecular and genetic mechanisms. However, the heterogeneous nature of the observed phenotypes reflects complex interactions between genetic, epigenetic, and environmental influences. This range of potential etiologies is more in line with the definition of a syndrome than with that of a disease. Understanding a “syndrome”, such as PCOS, begins with identifying the core mechanisms and selective factors responsible for simultaneous clustering of its clinical signs and symptoms. Based on the current consensus guideline, PCOS is diagnosed when at least two of the following criteria are met: (1) clinical or biochemical hyperandrogenism, (2) ovulatory dysfunction, and (3) polycystic ovarian morphology on ultrasound or raised levels of anti-Müllerian hormone (AMH) (6). Most recently, in a landmark global consensus, PCOS was renamed Polyendocrine Metabolic Ovarian Syndrome (PMOS) to capture its complex endocrine, metabolic, and ovarian pathophysiology (7). Recent research on PMOS has broadened our understanding of the potential initial mechanisms underlying this condition. Although genetic studies have explained only a small fraction of the syndrome, recent epigenetic discoveries hold considerable promise in accounting for the majority of PMOS cases. PMOS can be viewed as an intergenerational evolutionary adaptation to lifestyle and environmental influences, integrating genetic effects during fetal development, intergenerational inheritance, and epigenetic markers. In this review, we investigated the hypothesis that PMOS primarily originates during fetal development and assessed how stressors during this time might affect the subsequent phenotype. We propose that puberty is a critical period during which physiological changes and environmental factors alter the gut microbiome and interact with existing epigenetic marks, leading to the expression of the PCOS phenotype and related metabolic features. We also examined how these biological mechanisms integrate with the existing diagnostic criteria for PMOS. Our goal is to provide an integrated approach to the developmental origins, genetics, and epigenetics of this syndrome, thereby establishing the biological foundation that anticipates and supports the paradigm-shifting renaming to PMOS.

## Developmental plasticity and epigenetics

The fetus is most vulnerable to environmental influences during the embryonic period, when the fetus uses intrauterine stressors to adjust individual phenotype to the current and future predicted environment (8). This “developmental plasticity” explains how a normal genetic variant can produce variable phenotypes later in life (9). At the molecular level, developmental plasticity refers to an individual’s capacity to adjust gene expression in response to a ‘forecast’ of environmental conditions provided by the mother (8). This adjustment is thought to be mediated, at least in part, by epigenetic mechanisms (10). Epigenetic programming is widely regarded as a central, though not sole, contributor to developmental plasticity, given its capacity to modulate gene expression in response to the early-life environment and to persist into later life. However, gene regulation is a complex and interconnected process, involving the interplay between epigenetic mechanisms, transcription factors, signaling pathways, and other regulatory factors (11, 12).

## Developmental origin of health and disease and PMOS

Fetal development has been recognized as a “critical period” during which environmental exposures can induce long-lasting biological and physiological changes in tissue structure and function. Neel et al. (13) coined the “thrifty genotype hypothesis,” suggesting that intrauterine stressors, such as food scarcity, favor the selection of genes that enhance metabolic efficiency and energy storage, thereby conferring a survival advantage during periods of famine. However, these adaptations may become maladaptive at times of food abundance, predisposing individuals to metabolic disorders such as obesity and type 2 diabetes (T2D). Over time, evidence from longitudinal epidemiological studies suggests that fetuses exposed to famine at times of conflicts had lower birthweight and were more likely to develop diabetes and cardiovascular diseases later in life (14, 15). These landmark observations of Barker and Osmond set the foundation for the ‘fetal origins of health and disease’ hypothesis, highlighting that the developmental basis of chronic disease extends beyond genetic predisposition, as Neel had earlier proposed, to include the influence of intrauterine and early-life environmental factors (16).

The first formal hypothesis framing PMOS as the outcome of developmental programming was proposed by Abbott et al. in 2002 (17). The authors suggested that early, possibly prenatal, hyperandrogenism may program the Hypothalamic-Pituitary-Ovarian (HPO) axis and, together with genetic and metabolic factors, predispose the individual to PMOS. Numerous studies involving both animals and humans indicate that *in-utero* developmental programming of inherited genes constitutes the initial priming event, which is often referred to as the “first hit” (18, 19). This process may involve epigenetic modifications and alterations in gene expression patterns that could be established during fetal development. It is proposed that a ‘second hit’ can be potentially triggered by postnatal environmental factors and the onset of puberty. During these critical periods, the underlying genetic vulnerabilities that had potentially been established *in utero* may be unmasked (20, 21). The diverse range of phenotypes observed in PMOS may point to a complex interaction between genetic and environmental factors. To gain a firm grasp of PMOS pathophysiology, it is essential to disentangle the relative impact of these elements. Although direct mechanistic links between genotype and phenotype are not always feasible, current evidence suggests that genetic variation does not fully account for phenotypic diversity, pointing instead to shared biological pathways (22, 23). To tackle this complexity, the syndrome can be viewed as a progressive disorder with distinct phases, the first of which is influenced by the interplay between inherited genetic factors and the intrauterine environment.

## *In utero* environment: fetal development as a critical period

The germline represents the inherited genetic framework, established through the transmission of parental alleles, recombination, and imprinting. During fetal development, this genetic liability is dynamically modulated through stage-specific developmental programming and can be influenced by intrauterine exposures via epigenetic mechanisms (24). Teratogenicity and long-term oncogenic effects are examples of developmental programming due to epigenetic mechanisms, as demonstrated by diethylstilbestrol (DES) exposure from the late 1940s to the early 1970s. *In utero* DES exposure caused vaginal clear cell adenocarcinoma in young women decades after exposure (25). The teratogenic effect was linked to hypermethylation of the *HOXA10* gene, a key regulator of uterine organogenesis, which disrupted normal reproductive tract development (26). Substances that interfere with the normal functioning of the hormone system are collectively known as endocrine disturbing chemicals (EDCs) (27). Several EDCs have been shown to induce PMOS-like phenotype such as insulin resistance, hyperandrogenism, and systemic inflammation (28, 29). Bisphenol A (BPA) is a high-volume industrial chemical widely used in the production of polycarbonate plastics and epoxy resins. Recent epidemiological studies have reported higher body fluid BPA concentrations in women with PMOS compared with controls (30). BPA has also been linked to hyperandrogenism, insulin resistance, and other metabolic features of PMOS (31). Experimental studies in rodent models suggest that BPA exposure may influence biological pathways implicated in PMOS, including ovarian steroidogenesis, hypothalamic–pituitary–gonadal axis function, metabolic homeostasis, inflammation, follicular development, and epigenetic regulation (32–36). However, the causal direction of the human cross-sectional association remains unresolved. Specifically, metabolic disturbances observed in PMOS patients, such as increased adiposity and altered hepatic metabolism, may promote the accumulation of some EDCs. Therefore, the observed higher EDCs concentrations in cross-sectional studies could result from altered distribution and elimination rather than from higher external exposure (37). This possibility of reverse causation points to the need for prospective designs to assess exposures before diagnosis, ideally during fetal life, and to distinguish developmental origins from metabolic consequences.

### Developmental plasticity of the ovary

The question of whether PMOS is a primary disorder of the ovaries, as initially conceptualized, has led to research focusing on prenatal exposures that primarily affect the ovaries. The most extensively studied priming factor was prenatal androgen exposure and its effect on ovarian function and morphology. Ovarian development sets up the number and quality of primordial follicles, the granulosa cell programming, and the balance of steroidogenesis genes (38, 39). This morphological and functional development is mediated by three master regulators, including *WNT4, FOXL2*, and *FIGLA* (40). Genetic variations or mutations in these genes could, plausibly, contribute to disturbed ovarian morphology and function observed in PCOS. Human studies of PMOS have observed that WNT ligand/receptor expression is altered (41), antagonists such as *SFRP4* are dysregulated (42), and downstream signaling in beta-catenin is depleted (43). However, most of these studies are small, tissue-specific, and cross-sectional, making their conclusions suggestive and limiting their ability to establish temporality. Animal models provide compelling evidence that prenatal androgen excess can induce both morphological and functional disturbances in female offspring, closely resembling features of PMOS. In the rhesus monkey, prenatal androgen exposure results in disordered ovarian function, impaired fertility, hypersecretion of luteinizing hormone, and insulin resistance, replicating the reproductive and metabolic features of the human syndrome (44). Exogenous testosterone in rat models also directly disrupts early ovarian follicular dynamics and produces the disordered folliculogenesis characteristic of the polycystic ovary (45).

Maternal hyperandrogenemia may not be sufficient to directly program PMOS in female offspring, as the human fetus is partially protected from maternal androgen excess through placental aromatization of androgens to estrogens and the high concentrations of sex hormone–binding globulin during pregnancy (46). An alternative, or complementary, pathway may involve the maternal metabolic disturbances that frequently associate PMOS. Maternal obesity, insulin resistance, and hyperglycemia may alter the placental metabolic and endocrine environment and, in genetically susceptible female fetuses, contribute to fetal hyperinsulinemia (**Figure 1**) (47). This hyperinsulinemic state may promote ovarian androgen production and disrupt folliculogenesis *in utero* (47, 48). Evidence from animal models suggests that the timing of androgen exposure is a factor in the effect. In prenatally androgenized rhesus monkeys, exposure during critical periods, i.e., early to mid-gestation of fetal development, may produce more pronouncedovarian and PMOS-like phenotypes than exposure later in gestation (44). Moreover, androgen-induced maternal and fetal metabolic disturbances, such as obesity and transient hyperglycemia, may further compound this developmental plasticity (49). These findings support the concept that both the nature of the intrauterine exposure and its timing may influence the extent of reproductive and metabolic programming. In humans, evidence of excess prenatal androgen exposure comes from anatomical and biochemical markers, though both carry limitations. Observational studies have suggested that PMOS and elongated anogenital distance may be associated, indicating that it could be used as a clinical sign of hyperandrogenism exposure (50–54). In contrast, biochemical evidence is still inconclusive. Studies of cord blood androgens in daughters of women with PMOS indicate either higher, lower, or unchanged concentrations (50–52). Importantly, cord blood obtained at delivery may not accurately reflect androgen exposure during the critical period for programming ovarian development, i.e., early to mid-gestation. Moreover, it is still technically and ethically challenging to directly measure fetal steroid levels *in vivo* (55). It is true that anogenital distance provides a non-invasive marker of prenatal androgen exposure. However, its interpretation is limited by variability between populations, potential confounding by birthweight and growth patterns, as well as methodological differences in measurement (54, 56).

**Figure 1:**
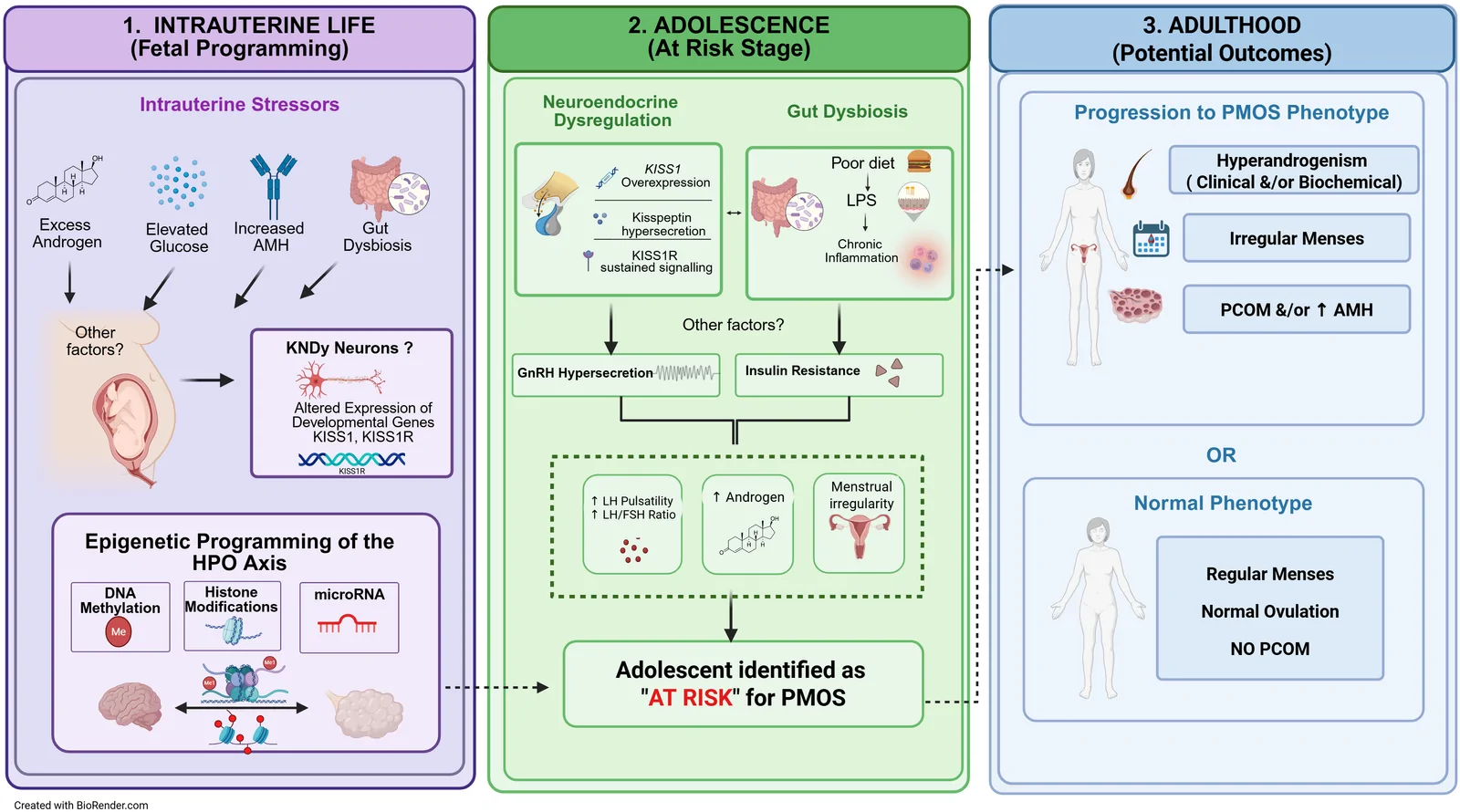
Schematic hypothesis of the developmental origins, genetics, and epigenetics of PMOS across three critical developmental periods. During intrauterine life, fetal exposure to excess androgens, elevated glucose levels, increased AMH, and gut dysbiosis may directly program the neuroendocrine and reproductive axes by epigenetically modulating the KNDy neurons and the expression of key developmental genes, especially KISS1. Alternatively or concurrently, maternal metabolic disturbances, such as obesity and insulin resistance, may alter the placental function and fetal hormonal and metabolic milieu. During adolescence, developmental programming can present as altered KNDy signaling, increased GnRH pulsatility, hyperandrogenism, and menstrual irregularity. Simultaneous environmental and dietary exposures may induce gut dysbiosis, inflammation, and insulin resistance, thereby further elevating ovarian androgen production and disrupting folliculogenesis. These factors collectively identify individuals “at risk” for PMOS. Progression into adulthood may subsequently lead to the full PMOS phenotype, or alternatively, some individuals may not progress to overt disease and remain phenotypically normal. PMOS can be viewed as a “conditional phenotype” where normal genetic variants have been programmed under certain intrauterine stressors to prime the fetus to respond to suspected postnatal exposures. However, when the extrauterine environment is unexpectedly altered, PMOS can manifest as a phenotype. AMH, Anti-Müllerian Hormone; FSH, Follicle-stimulating hormone; GnRH, gonadotropin-releasing hormone; HPO axis, hypothalamic-pituitary-ovarian axis; KNDy, Kisspeptin-Neurokinin B-Dynorphin; LH, Luteinizing Hormone; LPS, lipopolysaccharide; KISS1, kisspeptin-1 gene; KISS1R, KISS1 receptor; PCOM, polycystic ovarian morphology; PMOS, polyendocrine metabolic ovarian syndrome. This figure was created using BioRender, https://biorender.com.

## Reconsidering the ovary within the broader hypothalamic–pituitary–ovarian axis

A more plausible framework is to view the ovaries as part of the wider hypothalamic–pituitary–ovarian (HPO) axis. In this perspective, the ovaries respond to higher hormonal regulatory centers while exerting feedback effects on higher regulatory centers and a wide range of metabolic and endocrine effects on different tissues, in accordance with the recent renaming of PCOS to PMOS (7).

### Central regulation: GnRH and the KNDy network

Pulsatile release of GnRH from the hypothalamus is the primary stimulus for pituitary gonadotrophs (57). The frequency and amplitude of GnRH pulses regulate the secretion of luteinizing hormone (LH) and follicle-stimulating hormone (FSH) from the anterior pituitary. GnRH release is regulated by a network of hypothalamic neurons and metabolic signals, with the kisspeptin–neurokinin B–dynorphin (KNDy) system playing a central role (57). KNDy neurons are situated in the arcuate nucleus in animals and the infundibular nucleus in humans. These neurons produce kisspeptin and neurokinin B; however, the co-expression of dynorphin is well documented in animals, whereas its occurrence in humans is less clear (58). The classical KNDy hypothesis proposed a sequential mechanism, beginning with neurokinin B (NKB), which initiates the process; kisspeptin then propagates the signal; and dynorphin terminates the GnRH pulses (57, 58). Recent findings, however, indicate that the system is part of a larger, interconnected network, with KNDy neurons integrating additional regulatory factors and neurotransmitters (58). Studies in mice have demonstrated that blocking AMPA glutamatergic receptors reduces LH pulse frequency, indicating that NKB is not the sole excitatory factor (59). Comparable effects on LH pulsatility have been observed in rats and sheep following micro-injection of kisspeptin antagonists into the arcuate nucleus, supporting the role of kisspeptin in pulse generation. Research also indicates that dynorphin-mediated pulse termination is more complex and species-dependent than previously understood (60). Additional studies in mice have identified non-canonical Kiss1R populations in the arcuate nucleus that may further amplify pulses (61). In short, whereas the conventional model suggested that KNDy neurons generated synchronized GnRH/LH pulses via autoexcitatory and autoinhibitory mechanisms, current evidence leans toward a more integrated network that incorporates a range of neuronal, hormonal, and metabolic inputs (58).

KNDy neurons also provide a major interface between gonadal steroid feedback and central reproductive control (**Figure 1**). KNDy neurons express receptors for estrogen, progesterone, and androgen, which receive sex steroid hormonal feedback to regulate pulsatile kisspeptin secretion. Estrogen receptors in the arcuate KNDy neurons are involved in the negative feedback regulation of estrogen to the HPO axis (62). Contrarily, kisspeptin neurons in the rostral periventricular/preoptic region participate in the positive estradiol feedback that generates the preovulatory LH surge (63). This feedback loop is particularly relevant to PMOS. The increased GnRH/LH pulsatility observed in PMOS may indicate defective steroid feedback rather than just an intrinsically overactive GnRH generator. Experimental studies using a prenatal androgen-treated mouse model of PMOS showed elevated KNDy-cell androgen receptor expression but reduced progesterone receptor and dynorphin gene expression (64). These observations suggest that hyperandrogenism may directly alter the sensitivity of the HPO axis to steroid feedback (**Figure 1**). In healthy women, estradiol and progesterone dampen LH pulsatility by roughly 60%, but in women with PMOS this feedback is much weaker (65). This blunted response appears to be driven, at least in part, by the central effects of excess androgens, since blocking androgen receptors with flutamide has been shown to restore normal feedback sensitivity (65). Notably, conditional deletion of progesterone receptors from kisspeptin neurons in female mice does not impair progesterone-mediated suppression of LH pulses (66). Therefore, evidence points to impaired ovarian hormone feedback as one potential pathway, yet the mechanisms regulating GnRH pulsatility are more intricate and extend beyond changes in sex steroid receptor expression alone.

Findings from rodent and sheep models suggest that KNDy neurons detect and transmit information about energy status to GnRH neurons, alongside conveying gonadal steroid signals (67). Studies in ovariectomized, feed-restricted lambs show that undernutrition suppresses LH secretion primarily by reducing kisspeptin signaling within KNDy neurons (68). Administration of kisspeptin in undernourished rodent models restores gonadotropin secretion (69). These results indicate that KNDy neurons may show developmental plasticity that integrates early hormonal and metabolic environments into the reproductive axis, thereby modifying the subsequent responsiveness of the reproductive axis to nutritional and endocrine cues (**Figure 1**). Although this developmental mechanism is mainly backed by animal studies, it provides a plausible link among early-life environmental exposures, neural developmental plasticity, and the later emergence of the reproductive and metabolic phenotype associated with PMOS.

## The role of anti-Müllerian hormone

Animal models have shown that administration of AMH to pregnant mice increased maternal LH-driven testosterone secretion and produced PCOS-like features in adult female offspring (70). These offspring exhibited hyperactivated GnRH neurons, indicating that prenatal endocrine perturbations can reprogram hypothalamic and pituitary function, secondarily disrupting ovarian physiology. Elevated AMH levels in women with PMOS, including during pregnancy, suggest that this hormone may modify both maternal and fetal HPO signaling (47, 70). In addition, rare variants in *AMH* and *AMHR2* that impair signaling activity highlight genetic heterogeneity and support the view that multiple AMH-related pathways converge on HPO axis dysfunction (71). AMH is secreted by granulosa cells of small antral follicles and serves as a reliable marker of functional ovarian reserve (72). In adults, meta-analyses show pooled sensitivity of 0.79 and specificity of 0.87 for AMH as a diagnostic marker for PMOS, values comparable to those achieved by ultrasound defined Polycystic Ovarian Morphology (PCOM) (6). Therefore, AMH is currently used as an alternative to ultrasound-based PCOM in diagnostic criteria in adults. Besides its clinical use, genetic studies advance understanding of the biological relationship between AMH and PMOS. Large-scale GWAS meta-analyses demonstrate that PCOS risk alleles are frequently associated with higher circulating AMH levels. Five PMOS loci show nominal associations with AMH, and several overlap with loci that influence age at natural menopause (73, 74).Although these associations do not confirm that PMOS risk variants directly determine the establishment or maintenance of the follicle pool, the findings suggest that shared genetic pathways may link PMOS susceptibility with ovarian follicular dynamics and reproductive lifespan. In adolescence, however, AMH is not currently endorsed to diagnose PMOS due to a considerably lower performance, with pooled sensitivity and specificity of 0.66 and 0.78, respectively (6). The physiological overlap between normal pubertal maturation and early PMOS features may contribute to the observed variability between adults and adolescents. However, longitudinal evidence indicates that AMH levels display remarkable intra-individual stability from infancy to adolescence in normal girls. HPO axis is transiently activated during infancy, leading to detectable AMH levels with a peak at 3.5 months of age, before the HPO returns to a quiesce stage in mid-childhood (75, 76). Longitudinal analysis revealed that elevated mid-childhood AMH levels were positively associated with higher adolescent LH concentrations, an increased LH/FSH ratio, elevated testosterone levels, and a greater total follicle count, consistent with endocrine and ovarian features characteristic of PMOS (77). On the other end of the spectrum, among girls with Turner Syndrome (45,X), AMH concentrations are typically undetectable, whereas those with a mosaic karyotype exhibit measurable levels that positively correlate with the onset and progression of puberty (78).

### The gut–inflammation–insulin resistance axis in PMOS

Early studies linked PMOS with insulin resistance and noted a familial clustering of insulin secretion defects and associated metabolic disturbances (4, 79). Recent epidemiological studies show that about 70% of women with PMOS have clinically measurable insulin resistance, independent of body mass index (BMI) (80). Insulin acts through two main signaling pathways: metabolic and mitogenic. In the metabolic pathway, insulin binds to its receptor in skeletal muscle and in adipose tissue, triggering the phosphatidylinositol 3-kinase (PI3K)–Akt/PKB pathway. This results in GLUT4-mediated glucose uptake and inhibition of glycogen synthase kinase 3 (GSK3), thereby promoting the synthesis of glycogen, fatty acids, and proteins (81). On the other hand, the mitogenic pathway involves the translocation of ERK1/2 to the nucleus and the activation of transcription factors that control cellular growth, proliferation, differentiation, and gene expression in insulin-responsive tissues, such as the ovaries and the endometrium (81). Women with PMOS generally have a form of selective insulin resistance characterized by impaired metabolic signaling in skeletal muscle and in adipose tissue, together with a compensatory hyperinsulinemia and metabolic dysregulation. By contrast, the ovaries remain relatively sensitive to increased insulin signaling, which in turn stimulates theca-cell androgen production and leads to hyperandrogenism (81). From an evolutionary point of view, insulin resistance might be seen as an adaptive metabolic response rather than simply as a pathological abnormality (19). By selectively reducing insulin-mediated metabolic signaling, metabolism is shifted from an anabolic, energy-storing state to an energy-releasing state. The transition to a catabolic state increases the availability of glucose and free fatty acids to vital organs such as the brain (82). This adaptive mechanism would have been especially relevant during times of starvation, pregnancy, lactation, and adolescence, when competing demands for growth and reproduction are dominant (19). In this regard, the selective insulin resistance and the metabolic features of hyperinsulinemia and hyperandrogenism seen in women with PMOS may have provided advantages during periods of environmental or physiological stress that required greater metabolic demands. However, these adaptations could become maladaptive in contemporary environments where excess energy and sedentary behavior prevail.

The mechanisms by which modern environmental factors, including contemporary lifestyle, may unmask the PMOS phenotype in susceptible women have been the subject of investigation. The microbiome provides a plausible causal pathway that links the metabolic and reproductive manifestations of PMOS with lifestyle factors (**Figure 1**). The term “gut microbiome” encompasses all microorganisms residing in the gastrointestinal tract that contribute to host metabolism, immune regulation, maintenance of the intestinal barrier, and endocrine signaling via microbial metabolites (83). The gut microbiota ferments fiber-rich diets to produce organic acids such as acetic acid, propionic acid, butyric acid, and valeric acid. These metabolites, collectively referred to as short-chain fatty acids (SCFAs), mediate essential metabolic functions of the gut microbiota, regulate epithelial barrier integrity, modulate immune responses, and influence systemic metabolism through evolutionarily conserved mechanisms (84). SCFAs bind to specific G protein-coupled receptors in intestinal, adipose, skeletal muscle, liver, and pancreatic tissues, indicating a broader role for SCFAs in communication between the gut and peripheral tissues. SCFAs activate intracellular signaling pathways that promote glucagon-like peptide 1 (GLP-1) secretion within the intestine. In humans, duodenal administration of the butyrate-producing bacterium *A. soehngenii* L2-7, a source of microbial-derived SCFAs, has been shown to increase GLP-1 levels and improve insulin sensitivity (85). Several rodent studies have also demonstrated that both exogenous and microbiota-derived SCFAs can exert beneficial effects on the function and metabolism of tissues expressing SCFA receptors (86, 87). GLP-1 enhances insulin secretion in a glucose-dependent manner and improves insulin sensitivity. GLP-1 receptor agonists are increasingly preferred for high-risk T2D patients, particularly those with cardiovascular disease, and more recently, for long-term obesity management.

Dysbiosis refers to a disruption of the gut microbiome and an imbalance in SCFAs-producing bacteria. Tremellen et al. (88) proposed the dysbiosis of gut microbiota theory as a mechanism linking the evolutionary model of PMOS to lifestyle factors. This theory suggests that a poor-quality diet and resulting gut dysbiosis weaken the integrity of the intestinal barrier, leading to metabolic endotoxemia, chronic low-grade inflammation, and impaired insulin signaling (88). These metabolic disturbances, such as insulin resistance and compensatory hyperinsulinemia, may increase ovarian androgen production and disrupt folliculogenesis, establishing a mechanistic link between gut dysbiosis and the reproductive features of PMOS (**Figure 1**).

A key event in dysbiosis is the disruption of intestinal barrier function and increased permeability, allowing bacterial products, especially lipopolysaccharide (LPS), to enter the circulation and trigger chronic inflammation. LPS activates Toll-like receptor 4 on immune cells, leading to the production of cytokines and chemokines such as IL-1β and TNF-α (89). Animal studies have confirmed this mechanistic pathway in PMOS models. In a letrozole-induced PMOS rat model, Zheng et al. (90) found that LPS administration significantly increased ovarian inflammatory markers, including IL-1β and TNF-α, and impaired reproductive function, as shown by fewer corpora lutea and more cystic follicles. Similarly, the dehydroepiandrosterone (DHEA)-induced PMOS mouse model showed compromised intestinal barrier integrity, decreased GLP-1 levels, higher serum LPS concentrations, and upregulation of the ovarian TLR4-NLRP3 inflammatory signaling pathway (91). These changes were reversed with dietary modification, suggesting a potential mechanistic role for epigenetic factors.

Observational studies have found that reduced microbial diversity, microbial imbalance, and altered microbial metabolic function are main characteristics of gut dysbiosis in women with PMOS. Torres et al. (92) examined the gut microbiome of 73 women with PMOS and 48 healthy controls and found that those with PMOS had significantly lower alpha diversity. This implies reduced microbial richness and a more uneven distribution of microbial groups in patients with PMOS. Higher total testosterone levels and the presence of hirsutism were also associated with reduced diversity in the gut microbiome. On the other hand, a recent systematic analysis revealed no significant difference in alpha diversity between women with PMOS and healthy controls, but it did identify distinct gut microbial composition and differences in several bacterial genera (93). The microbial differences were found to vary according to testosterone levels, which suggests a possible link between gut dysbiosis and the hyperandrogenic features of PMOS. In patients with PMOS, SCFAs are generally altered rather than simply reduced. Specifically, elevated levels of propionate have been linked to insulin resistance and other metabolic features associated with PMOS (94). Nevertheless, the variability in SCFA profiles, clinical phenotypes, and study methods hinders the ability to draw causal inferences or reach definitive conclusions (95).

Collectively, experimental and clinical evidence proposes that gut dysbiosis and resulting elevated circulating LPS, may promote systemic inflammation and contribute to insulin resistance in PMOS (**Figure 1**). Epigenetic mechanisms may represent one of the factors regulating dysbiosis-inflammation-insulin resistance axis (refer to Epigenetics and Gut Microbiome in PMOS). Other pathways have also been implicated (96). Nevertheless, the causal direction of this gut–inflammation–insulin resistance-ovarian axis in humans remains uncertain and requires further investigation.

## The genetic basis of PMOS

The inheritance basis of PMOS was first suggested by familial studies, which reported a 20–40% prevalence among first-degree relatives compared with background prevalence (97). The clustering of reproductive phenotypes, such as hyperandrogenemia, initially raised the possibility of a monogenic pattern of inheritance. Similarly, metabolic features of the syndrome, including obesity, T2D, and hyperinsulinemia have been identified among relatives (98). Together, these findings support a genetic susceptibility underlying PMOS, nevertheless the precise pattern of inheritance has long been a matter of debate. The realization that PMOS shows a spectrum of phenotypic variation have shifted the focus from monogenic factor to a more complex polygenic basis and environmental contribution. Understanding PMOS begins with determining the relative contribution of genetic factors to the phenotypic differences (i.e., heritability of PMOS) compared to environmental factors. Early twin studies estimated that genetic factors accounted for up to 79% of variation in PMOS (99). These estimates were likely inflated because they assumed that greater similarity in monozygotic compared to dizygotic twins reflected only genetic effects. In reality, monozygotic twins also share more similar intrauterine environments as well as postnatal lifestyle factors, both of which can contribute to phenotypic concordance independently of genetic variation.

Current genomic research on complex traits has shown a definitive move toward GWAS to account for the cumulative effects of common variants on shaping complex traits, including PMOS. The first two GWAS studies were conducted in China and identified 11 risk loci associated with PMOS, two of the loci were subsequently replicated in patients with European ancestry (100–102). Most, but not all, identified loci contribute to PMOS through one of three mechanisms: (1) metabolic pathways (*THADA, INSR, HMGA2*); (2) hormonal regulation including through the HPO axis (*FSHB*, *LHCGR*, *FSHR*, *DENND1A*); and (3) the oocyte/follicle complement (*HMGA2, YAP1).* Of note, GWAS identifies loci rather than genes, thus creating biological uncertainty and necessitating functional mapping to link loci to causal genes and pathways. Moreover, identified loci can only explain up to 10% of PMOS heritability, suggesting that other factors such as epigenetics, may play a stronger role in PMOS pathogenesis (103). The most recent GWAS meta-analysis has identified 29 independent loci associated with PMOS across ancestries (74). The fact that many susceptible loci are shared across various ancestries supports the notion of the evolutionary origin of PMOS. Genetic variations observed in PMOS arise from naturally occurring random mutations in the germline, then transmitted across generations and distributed among populations through drift, migration, and selection (104). PMOS variants that impact metabolic, hormonal, and ovarian pathways, as demonstrated in GWAS studies, can offer a survival advantage in certain conditions, but become disadvantageous when environmental exposures change (105). Accordingly, PMOS can be viewed as a “conditional phenotype”, where normal genetic variants have been programmed under certain intrauterine stressors to prime the fetus to respond to suspected postnatal exposures. However, when extrauterine environment is unexpectedly changed, PMOS appears as a phenotype (**Figure 1**).

### Epigenetics of PMOS

Epigenetics is believed to be the mechanism that explains how intrauterine stressors can lead to PMOS phenotypes in female offspring. Parental preconception factors, including nutritional imbalance, hyperglycemia, obesity, androgen excess, and gut dysbiosis, can induce germline epigenetic modifications that potentially mediate the intergenerational transmission of cardiometabolic and reproductive traits (106, 107). Experimental models provide supporting evidence that alterations in oocyte transcriptional and mitochondrial function, ovarian DNA methylation, and sperm-derived small noncoding RNAs could be linked to the development and inheritance of PMOS in humans (103, 106, 108). For years, PMOS was primarily attributed to hormonal and metabolic disturbances, hyperinsulinemia, chronic inflammation, and oxidative stress. Increasing evidence suggests that epigenetic mechanisms, including DNA methylation, histone modifications, and non-coding RNAs, may contribute to disease development by regulating genes involved in steroidogenesis, insulin signaling, inflammation and gut dysbiosis (109, 110).

#### Epigenetic regulation of PMOS onset and reproductive phenotype

The hallmark of puberty is the activation of HPO axis, which is primarily driven by increase in the pulsatile secretion of gonadotropin-releasing hormone (GnRH) from the hypothalamus. GnRH induces the pituitary gland to produce gonadotrophins (LH and FSH), which then activate sex steroidogenesis and gametogenesis in the ovaries. Although pulsatility of GnRH is the critical hormonal event in puberty, the exact trigger for its onset remains unclear. As discussed earlier, Kisspeptin and Nuerokinin act as potential upstream regulators of GnRH pulsatility. while Gamma-aminobutyric acid (GABA) and loss-of-function variant in the *MKRN3* gene may act as inhibitors (111–113). The role of Kisspeptin was suggested following the discovery that hypogonadotropic hypogonadism in humans can be caused by the loss-of-function variants in the *KISS1* and *KISS1R* genes, which encode the Kisspeptin neuropeptide and its receptor respectively (111, 114). Nevertheless, administration of Kisspeptin in animal models increased GnRH pulsatility.

Epigenetic regulation of *KISS1* gene is a key player in the initiation of puberty. The shift of *KISS1* from a repressed to an active state in the hypothalamic neurons enables GnRH pulsatility. The switch is complex and multifaceted phenomenon that is mediated by coordinated removal of Polycomb repressive complexes and activation of Trithorax group proteins, with additional modulation by nutritional and environmental cues (115). This epigenetic plasticity makes the *KISS1* system a plausible target of prenatal programming. At puberty, LH pulsatility becomes exaggerated in PMOS, which suggests that intrauterine exposures may prime the kisspeptin–GnRH network toward hyperactivity (116, 117). Abnormal GnRH pulsatility is associated with a characteristic elevation in the LH/FSH ratio seen in patients with PMOS (118). Elevated LH preferentially stimulates excessive androgen production in ovarian theca cells, whereas relatively low FSH fails to support normal follicular development (119). Consequently, these endocrine disturbances lead to the key reproductive hallmarks of PMOS, namely hyperandrogenism and anovulation. Experimental evidence from a mouse model with induced PMOS showed that suppressing hyperactive kisspeptin neurons reversed the abnormally high LH pulse secretion and hyperandrogenemia (120). Although there is currently no direct evidence demonstrating that prenatal exposures induce specific KISS1 chromatin alterations in the human hypothalamus that subsequently contribute to PMOS, evidence from experimental animal studies supports a role for epigenetic regulation of the kisspeptin–GnRH network in reproductive neuroendocrine function. Direct characterization of KISS1 chromatin states in the human hypothalamus remains inherently challenging because of the limited accessibility of hypothalamic tissue, particularly during developmental stages. Thus, this experimental evidence provides a plausible mechanistic framework in which epigenetic regulation of the Kiss1 gene contributes to estrogen-positive feedback that drives the GnRH/gonadotropin surge in brain mice. This suggests that epigenetic alterations established *in utero* could remain latent until the pubertal activation of the HPO axis, potentially acting as a developmental “second hit” that contributes to the hyperactivation of the kisspeptin-GnRH network and the reproductive disturbances characteristic of PMOS (**Figure 1**) (121, 122).

#### Epigenetic regulation of metabolic and endocrine dysfunction in PMOS

Metabolic dysfunction in PMOS could be likely driven by complex epigenetic modifications that regulate gene networks involved in glucose and lipid metabolism. Differential methylation analysis (adjusted *p* ≤ 0.05) identified 185 hypermethylated and 887 hypomethylated regions in ovaries from PAMH F3 offspring mice (third-generation progeny of AMH exposure) (123). Integrated methylation and transcriptomic analysis identified genes, such as *Robo1, Sorbs2, Cdkn1a*, and *Hdc*, involved in lipid metabolism, insulin signaling, inflammation, and T2D pathways (123). Altered pathways also included cell cycle regulation and Notch signaling, further supporting a role for epigenetic dysregulation in the metabolic abnormalities of PMOS (123). The role of epigenetics in metabolic disturbances has also been replicated in human studies. In women with PMOS, differentially methylated regions (DMRs)in whole blood samples were enriched in genes and biological pathways involved in triglyceride biosynthesis, lipid metabolism, thyroid hormone signaling, and metabolic regulation (124). Several DMRs overlapped with established PMOS susceptibility genes, including *THADA, AOPEP*, and *TRIML2* (124). For instance, *THADA* gene plays a role in death receptor pathway and apoptosis, and has been identified as being related to PMOS development and T2D in GWAS (101). These findings suggest that epigenetic dysregulation may contribute to the development of insulin resistance, obesity, dyslipidemia, and other metabolic abnormalities characteristic of PMOS (**Figure 1**).

Emerging evidence suggests that the metabolic-related epigenetic dysregulation in PMOS is potentially reversible through lifestyle interventions (125). In animal models, treadmill exercise and/or alternate-day feeding, have been shown to downregulate the expression of *DNMT1* and *DNMT3B* (126). These changes in chromatin modifier enzyme lead to DNA hypomethylation and can improve glucose tolerance and ovarian function (127, 128). Moreover, lifestyle changes can modulate histone acetylation through changes in *HDAC1* and *HDAC2* expression, which is associated with decreased weight gain and metabolic balance (126, 129). The ability of physical activity and caloric restriction to influence the expression and activity of epigenetic-modifying enzymes highlights the potential for lifestyle interventions to reshape epigenetic regulation and underscores the importance of managing maternal conditions before and during pregnancy to break the cycle of intergenerational metabolic disease. Current International Evidence-based Guideline for the assessment and management of polycystic ovary syndrome 2023 endorses healthy lifestyle behaviors, including dietary modification and regular exercise, in all women with PMOS (6). While these recommendations are chiefly intended to enhance metabolic health, reproductive outcomes, and the quality of life for affected females, their advantages could extend beyond the individual to include offspring and future generations.

Methylation patterns potentially mediating the epigenetic regulation of metabolic and reproductive pathways in offspring have been investigated. Echiburú et al. found that DNA methylation patterns were altered in the offspring of women with PMOS (130). Specifically, promoter methylation of reproductive and metabolic genes in whole-blood DNA from infants (2–3 months old) differed between offspring of women with PMOS, controls, and those exposed to metformin during pregnancy. Despite the relatively small sample size (n = 48), both male and female offspring exhibited sex-specific differences in CpG methylation within key genes, including as leptin (*LEP*), leptin receptor (*LEPR*), adiponectin (*ADIPOQ*), adiponectin receptor 1 and 2 (*ADIPOR1*, *ADIPOR2*), *AMH*, and the androgen receptor (*AR*) genes (130). More recently, Polinski et al. examined the newborn DNA methylation among mothers with PMOS and hirsutism, in two different cohorts, the Upstate KIDS and the Effects of Aspirin in Gestation and Reproduction (EAGeR) cohorts, and they suggested that excess circulating maternal androgens may alter the DNA methylation of offspring in infancy (131).

### Epigenetics and gut microbiome in PMOS

Histone modifications are reversible chemical changes to histone proteins that modulate chromatin accessibility and regulate gene expression (109). Histone deacetylases (HDACs) are epigenetic enzymes which remove acetyl groups from the histone tails, a chemical modification that usually reduces gene accessibility. Among HDACs, histone deacetylase 3 (HDAC3) has been implicated in the transcriptional regulation of inflammatory and metabolic pathways associated with insulin resistance, including those relevant to T2D (132). HDAC3 may therefore serve as a molecular interface linking altered microbial metabolites, metabolic stress, inflammation, and dysregulated insulin signaling in PMOS (109). As described earlier, women with PMOS tend to have altered availability of SCFAs. Among SCFAs, butyrate acts as a natural HDACs inhibitor (109, 133). In obese individuals, sodium phenylbutyrate, a butyrate derivative with HDAC-inhibitory activity, has been shown to reduce lipid-induced insulin resistance, suppress endoplasmic reticulum stress, and protect pancreatic β-cell function (134). Additionally, butyrate may influence epigenetic processes by regulating mRNA N6-methyladenosine (m6A) methylation. Mechanistically, butyrate has been shown to inhibit the m6A methyltransferase METTL3, resulting in reduced expression of *FOSL2*, a regulator implicated in inflammatory responses (135) A recent study in rats demonstrated that PMOS induced by a diet high in fat and sugar (DHFD) was associated with epigenetic dysregulation. This dysregulation included increased activity of DNA methyltransferases DNMT1 and DNMT3A, as well as HDAC3, enhanced H3K9 methylation, decreased H3K9 acetylation, and gene-specific methylation changes affecting steroidogenesis and insulin signaling (136). These epigenetic alterations were accompanied by gut dysbiosis. Interventions with galacto-oligosaccharides (administered as a probiotic) and choline partially restored both the gut microbiome and epigenetic profiles, with folic acid identified as a potential molecular mediator. Furthermore, intergenerational transmission of epigenetic changes was observed, particularly increased DNMTs and HDAC3 activity and altered histone marks in F1 offspring (136). Taken together, mounting evidence suggests that the gut microbiome-metabolite-epigenetic axis may contribute to ovarian inflammation and metabolic abnormalities of PMOS.; yet the majority of the mechanistic evidence is still based on preclinical studies and not causal in humans (**Figure 1**).

## Current PMOS guidelines and the timing of epigenetic manifestation

Physiologically, the first year following menarche is characterized by anovulation in approximately half of menstrual cycles. However, most irregular cycles become ovulatory within two years after menarche (137). The exact timing for irregular cycles to suggest ongoing menstrual dysfunction is still not clear (138). The current guidelines recognize the two facets of overdiagnosis and the psychological and clinical implications of delayed diagnosis among adolescents (139). Therefore, the recommendations aim to balance these two extremes by identifying girls with clinical features suggestive of PMOS (i.e., either menstrual irregularity or hyperandrogenism) but who do not yet fulfill the diagnostic criteria as “at risk” (**Figure 1**) (6). Those high-risk females are advised to undergo reassessment at the time of reproductive maturation (6). The guideline suggests waiting for 8 years post-menarche to align with the typical time required for the HPO axis to mature fully. This period spans from the average age of menarche (i.e., 12 years old) to the end of adolescence (i.e., around 19 years old). By this stage, reproductive maturation is expected to be established, allowing more reliable clinical assessment of folliculogenesis, hormonal profiles, and ovulatory function.

Supporting this approach, longitudinal studies have shown that the predictive value of many PMOS-like features in adolescent girls, particularly elevated AMH, PCOM, and biochemical hyperandrogenism, is limited. Adolescents with oligomenorrhea are approximately 4.4 times more likely to develop PMOS in adulthood compared to those with regular menstrual cycles (140). Those adolescents with oligomenorrhea could, therefore, be labeled as “at risk” in accordance with current guidelines (6). However, the predictive value of isolated elevated AMH or the presence of PCOM is less than 22%, making them weak predictors. Even when these features coexist, the inclusion of high AMH in adolescent girls with oligomenorrhea to predict the risk of developing PMOSin adulthood is not effective (140). These findings highlight that many PMOS-like features in adolescence reflect physiological development rather than pathological process. Therefore, the rationale for delaying the definitive diagnosis of PMOS until after full HPO axis maturation, which is typically eight years post-menarche, is supported.

The question of whether puberty acts as a second critical epigenetic period, or merely a manifestation phase of pre-programmed epigenetic marks is still largely unanswered. The onset of puberty is a uniquely dynamic neuroendocrine transition period characterized by activation of GnRH pulsatility, induction of gonadal steroidogenesis, physiological insulin resistance, and adipose remodeling (141). These fundamental developmental changes of puberty may serve to unmask epigenetic marks established during *in utero* development, thus acting as a second hit (18, 19). Nevertheless, accumulating evidence also indicates that puberty retains a degree of epigenetic plasticity. Experimental studies demonstrate that environmental stressors, such as psychological distress, obesity, and eating disorders during adolescence can induce *de novo* epigenetic modifications (142). For instance, excessive nutrition in rodents induces a permissive chromatin state in hypothalamic Kiss1 neurons, which leads to accelerated pubertal activation of the reproductive axis (143). The HPO axis and adipose tissue interaction is orchestrated by Leptin, a hormone secreted from adipocytes that regulates KNDy neuron activity, Kisspeptin expression, and GnRH pulsatility in response to metabolic status (144–146). Persistent hypothalamic leptin resistance may develop in obese adolescents through epigenetically mediated upregulation of suppressor of cytokine signaling 3 (SOCS3), resulting in inhibition of hypothalamic leptin receptor signaling (147, 148). When leptin resistance develops, the hypothalamus becomes refractory to leptin signaling, leading to leptin overproduction and a vicious-cycle of dysregulated appetite and energy balance. Dysregulated GnRH pulsatility preferentially favors LH secretion at the expense of FSH, thereby promoting theca cell hyperandrogenism and contributing to chronic anovulation (119). Concurrently, leptin resistance at the level of the ovaries impairs granulosa cell function and subsequently leads to defective follicular maturation, follicular arrest, and elevated AMH production (149, 150). AMH has a central effect by increasing the neuronal firing frequency of GnRH neurons, while it has a peripheral effect on the ovaries by reducing follicular sensitivity to FSH (151). Together, leptin resistance initiates and reinforces a pathogenic circuit that sustains both the neuroendocrine and ovarian components of PMOS. This epigenetically programmed pathological triangle between leptin resistance, AMH excess, and GnRH dysregulation may predispose susceptible individuals to the development and long-term persistence of PMOS into adulthood.

## Synthesizing the unified evolutionary model: a conceptual framework

We propose a unified developmental - environmental model in which PMOS susceptibility may arise through the interaction of prenatal programming and later-life environmental exposures (**Figure 1**). The first hit may occur *in utero*, during which exposure to adverse maternal endocrine and metabolic disturbances, particularly maternal hyperandrogenism and other hormonal factors, can program genetic variants involved in the developing HPO axis and establish a latent susceptibility to PMOS. Epigenetic mechanisms may provide a mechanistic link between these early-life exposures and persistent alterations in phenotypes by modifying the expression of genes involved in metabolic, reproductive, and neuroendocrine pathways. Importantly, maternal hyperandrogenism may also influence the composition and function of the maternal microbiome, thereby potentially linking the prenatal endocrine environment to gut dysbiosis. The offspring acquires a considerable proportion of its early microbiome from the mother, particularly at birth and during nursing (152). This vertical transmission of a dysbiotic maternal microbiome may provide an additional mechanism through which maternal PMOS-related traits are propagated across generations. Thus, the first hit may not be limited to fetal exposure to an altered hormonal and metabolic environment but may also involve interactions among endocrine, metabolic, epigenetic, and microbial influences. This developmental susceptibility may subsequently be modified or unmasked by environmental exposures during later developmental periods, particularly puberty.

Puberty acts as a critical period during which physiological changes such as activation of the HPO axis, changes in insulin sensitivity and body composition, and increased exposure to dietary and lifestyle factors, place greater demands on neuroendocrine and reproductive systems (**Figure 1**). In susceptible individuals, these physiological stressors and adverse environmental exposures, such as a modern obesogenic diet, may disrupt the gut microbiome and induce dysbiosis. Obese adolescents with PMOS exhibit distinct gut microbial alterations that are associated with hyperandrogenism and metabolic abnormalities (153). Dysbiosis in adolescence may increase intestinal permeability to microbial metabolites, ultimately inducing systemic inflammatory pathways and insulin resistance (154). Compensatory hyperinsulinemia may further enhance ovarian androgen production, amplifying hyperandrogenism and progressively exposing the underlying reproductive phenotype. Epigenetic plasticity may facilitate the transition from a subclinical state of susceptibility to the overt PMOS phenotype. Accordingly, dysbiosis appears to mediate, rather than independently trigger, the environmental amplification of fetal-origin susceptibility, with epigenetic modifications serving as the underlying biological mechanism (**Figure 1**).

From an evolutionary perspective, this model may represent a form of ‘developmental mismatch’ theory (8). Developmental plasticity allows the fetus to respond to maternal environmental cues and adjust its physiology in anticipation of the postnatal environment; however, when the environment encountered later in life differs substantially from that anticipated during development, an adaptive mechanism may become maladaptive. PMOS could therefore be viewed as the phenotypic expression of a developmentally primed susceptibility when later environmental stressors, including dietary and lifestyle factors, exceed the individual’s metabolic and reproductive adaptive capacity. Within this framework, maternal health and the maternal microbiome constitute potential intergenerational determinants of susceptibility, whereas the postnatal environment determines, at least in part, whether that susceptibility remains compensated or progresses to overt PMOS. This also identifies preconception and pregnancy periods as potential windows for prevention, although whether modifying the maternal microbiome can reduce offspring PMOS risk remains to be established.

## Future directions

The methodological limitation in PMOS research is that it is overwhelmingly dependent on cross-sectional study designs, which, by definition, cannot determine the direction of causality for observed associations. Longitudinal studies are needed to establish temporality and infer causality. The most comprehensive systematic review of longitudinal PMOS research have identified only 21 longitudinal studies encompassing 62,123 participants across four continents, with follow-up periods ranging from 4 weeks to 32 years (155). The authors concluded that existing longitudinal data on changes in reproductive, psychological, and oncological features in PMOS are inadequate and conflicting, and the natural history of PMOS remains unclear, which limits prevention and early diagnosis strategies across the lifespan (155). The 2023 International Evidence-Based PCOS Guideline, along with its clinical research roadmap, acknowledges a significant gap in translating the genetic and epigenetic mechanisms of PMOS to clinical practice (156, 157). Recent genomic studies indicate shared biological mechanisms among different phenotypes of PMOS (23). However, there is still a lack of strong correlation between genetic data and clinical signs and symptoms. For this reason, the need for large longitudinal cohorts and international data repositories becomes essential to elucidate developmental pathways (156, 157).

What is still lacking, and what the field urgently requires, is a prospective longitudinal birth cohort study, ideally in daughters of women with PMOS. Epigenomic profiling of cord blood at delivery and peripheral blood during puberty is necessary for the study, as well as incorporating potential environmental stressors, including prenatal exposures, metabolic and hormonal profiles, AMH trajectories, psychological stress measures, and dietary patterns, among many other factors. Prospective adolescent-to-adult cohorts are needed to evaluate the International Evidence-Based PCOS Guideline recommendations for lifestyle factors and interventions as first-line treatment and establish their temporal relationships with subsequent metabolic and reproductive outcomes. This longitudinal study design is capable of determining the relative contributions, timing, and reversibility of prenatal versus peripubertal epigenetic programming in PMOS, and whether puberty primarily unmasks or promotes these epigenetic alterations.

## Conclusion

In this review, we present a comprehensive framework to clarify the multifaceted pathogenesis of PMOS. PMOS can be seen as a phenotype shaped by developmental factors arising from the interaction among genetic susceptibility, intrauterine programming, and postnatal environmental stressors. Prenatal hormonal and metabolic disturbances may prime the fetus through epigenetic and neuroendocrine programming of the KNDy–GnRH–ovarian axis. Subsequent nutritional, metabolic, inflammatory, endocrine, and microbiome-related exposures may further increase this vulnerability during adolescence and adulthood. The gut microbiome is now increasingly recognized as a potential mediator between environmental exposures and the metabolic and reproductive features of PMOS. Epigenetic mechanisms may provide the mechanistic link between early-life programming and later phenotypic expression. All these processes come together in an integrated PMOS network in which neuroendocrine, ovarian, metabolic, and inflammatory pathways interact bidirectionally. Developmental programming may establish susceptibility without inevitably resulting in clinical PMOS, while later environmental and metabolic factors determine phenotypic outcomes. The framework brings together the developmental, neuroendocrine, metabolic, inflammatory, microbial, and epigenetic components of PMOS and highlights the importance of conducting longitudinal human studies to establish causal relationships among these pathways.
